# To Remove Enamel Previous to Crowning

**Published:** 1898-04

**Authors:** H. H. Johnson


ARTICLE V.

### TO REMOVE ENAMEL PREVIOUS TO CROWNING.

BY H. H. JOHNSON, D. D. S.

I will give my method for the above operation, which is as follows :

Take a molar or bicuspid, for example, which is to be prepared for an all-gold shell crown. When spacing will be required between the tooth to be crowned and the adjoining tooth, it can be more easily accomplished with vulcan disks, which are thin, strong, flexible and cut rapidly. Care should always be used not to cut the opposite tooth, which can easily be avoided by using pressure towards the tooth to be prepared. By the time sufficient space has been obtained these two sides, the anterior and posterior approximal, have generally been squared up very well, as these thin disks will easily pass down below the festoon of gum and remove all the enamel.

The buccal and lingual sides and the approximo-buccal and approximo-lingual corners is generally where the difficulty arises.

The buccal and lingual sides down to within a few lines of the gingival margin may be easily and rapidly cut away with small carborundum wheels, which now leaves the crown of the tooth above the neck in a square shape. The disk has flattened it on the approximal sides, and the small wheels on the buccal and lingual sides. By taking small, thin, double convex carborundum wheels these comers may be cut down until the tooth takes a proper shape and all the enamel is removed, except a little ring around the neck of the tooth, which generally extends up just beneath the gum margin. To remove this little ring of enamel there is nothing so effectual as the vulcan or concavo-convex wheels. The thin edge of these little vulcan cup wheels will pass easily down below the margin of the gum without scarcely wounding it and cut off all the irregularities and excrescences. The range of work wbich these little wheels will do is remarkable, and they are hard and tough and do not wear easily. After cutting and leveling down the bumps, the surfaces of the crown should be planed off smooth by using scalers made very hard and sharp. For this purpose the ordinary diamond pointed, double-edged scalers are best.

By placing the thumb on the crown of the tooth and grasping the scaler firmly in the fingers, commencing at the gum line and pulling downward towards the cutting edge, the surfaces can be effectually planed off and rendered perfectly smooth. If any enamel should have been left by the wheels it can be easily removed with the scalers in this way.

As these scalers come from the manufacturers, they are too soft for the purposes suggested above, and must be hardened. This can easily be done by heating the point to a white heat and plunging in cold water or oil. Some claim that mercury is still better. After treating them in this way you have an instrument that will cut enamel, or even glass if required.

In those cases of leaning third molars which have tipped forward on account of the loss of the molars in front these little cup-shaped vulcan wheels are indispensable for reaching the anterior surface.

I shall offer no apology for consuming so much space in describing a simple method of removing enamel. I believe the importance of the subject justifies it.—*American Dental Weekly.*

